# An Unusual Complication of Lisinopril: A Report of Iatrogenic Henoch-Schönlein Purpura

**DOI:** 10.7759/cureus.68323

**Published:** 2024-08-31

**Authors:** Sónia Santos, Francisco Santos, Inês Fróis Cunha, Margarida Rato, Sofia Camões

**Affiliations:** 1 Internal Medicine, Unidade Local de Saúde Viseu Dão-Lafões, Viseu, PRT; 2 Cardiology, Unidade Local de Saúde Viseu Dão-Lafões, Viseu, PRT; 3 Dermatology, Unidade Local de Saúde Viseu Dão-Lafões, Viseu, PRT

**Keywords:** immunoglobulin a, leukocytoclastic vasculitis, lisinopril, hematuria, abdominal pain, henoch-schönlein purpura

## Abstract

Henoch-Schönlein purpura (HSP), also known as IgA vasculitis, is a hypersensitivity vasculitis characterized by palpable purpuric lesions associated with polyarthralgia, abdominal discomfort, and renal involvement. We present the case of a 41-year-old man who was admitted to the emergency department due to generalized purpuric lesions and abdominal pain. During the complementary study, there was no evidence of thrombocytopenia or coagulopathy but confirmed microscopic haematuria. The diagnosis of HSP was supported by the presence of leukocytoclastic vasculitis with perivascular IgA deposits in the skin biopsy. After excluding infectious, autoimmune, and neoplastic pathologies, the possibility of HSP associated with taking lisinopril, which had been recently initiated after hospitalization for acute heart failure, was assumed. Angiotensin-converting enzyme (ACE) inhibitor suspension and treatment with systemic corticosteroids lead to significant clinical regression, supporting our suspicion.

## Introduction

Henoch-Schönlein purpura (HSP) represents a systemic leukocytoclastic vasculitis characterized by perivascular deposition of IgA immunocomplexes in the small vessels [1] of the skin, gastrointestinal tract, joints, and renal glomeruli [2]. Although there is a higher rate of diagnosis in pediatric age, HSP represents the most prevalent vasculitis in adults, with an estimated incidence of 0.1 to 1.8 per 100,000 patients. The average age of presentation is 50, with a predominance in males [3].

Its pathogenic process is not fully known, although literature and clinical evidence support the importance of endothelial damage caused by deposits of IgA immune complexes in the vascular territory after induction of a pro-inflammatory state with activation and migration of neutrophils [4]. Clinically, HSP is suggested by the presence of the diagnostic tetrad: palpable purpura, polyarthralgia, and gastrointestinal and renal involvement [3]. Erythematous, macular, or urticarial skin eruptions are the most prevalent clinical manifestation observed in 75% of patients in the first 24 to 48 hours, commonly evolving to deep ecchymoses and/or palpable purpura with predominant distribution in the lower limbs [2,5]. In 66% of the patients, polyarticular involvement mainly of the knees and ankles resulting from synovitis is observed. Regarding the gastrointestinal tract, the spectrum of symptoms is broad, ranging from abdominal cramps to potentially fatal digestive hemorrhage due to the development of erosions, purpura, and necrosis of the digestive mucosa, preferentially located in the duodenum [5]. Renal involvement, which is commonly observed in adults, is characterized by the presence of proteinuria and/or haematuria associated with renal dysfunction with a risk of progression to end-stage renal disease, especially when associated with gastrointestinal manifestations and hemorrhagic skin lesions [3].

Henoch-Schönlein purpura should be considered in a patient with palpable purpura without thrombocytopenia or coagulopathy associated with the clinical manifestations listed above with evidence of leukocytoclastic vasculitis with immunofluorescence for IgA in the skin histopathological study [1]. Clinical suspicion is reinforced by the combination of clinical features and potential risk factors including upper respiratory tract infections which account for 50% of cases of this vasculitis (mostly caused by group A *Streptococcus*, adenovirus, parvovirus, *Mycoplasma pneumoniae,* and in rare cases *Helicobacter pylori*), allergic reactions, immunizations (Hepatitis B, influenza, yellow fever, SARS-COV2), drug iatrogenesis (antibiotics such as clarithromycin and quinolones, warfarin, chemotherapy drugs, immunomodulators, and in rare cases angiotensin-converting enzyme (ACE) inhibitors), inflammatory bowel disease, spondyloarthropathies and neoplasms of solid organs (lung, prostate, and kidney), and hematological cancers [1,3,5]. Here, we report a clinical case of HSP as a complication of a conventional treatment in clinical practice.

## Case presentation

**Figure 1:**
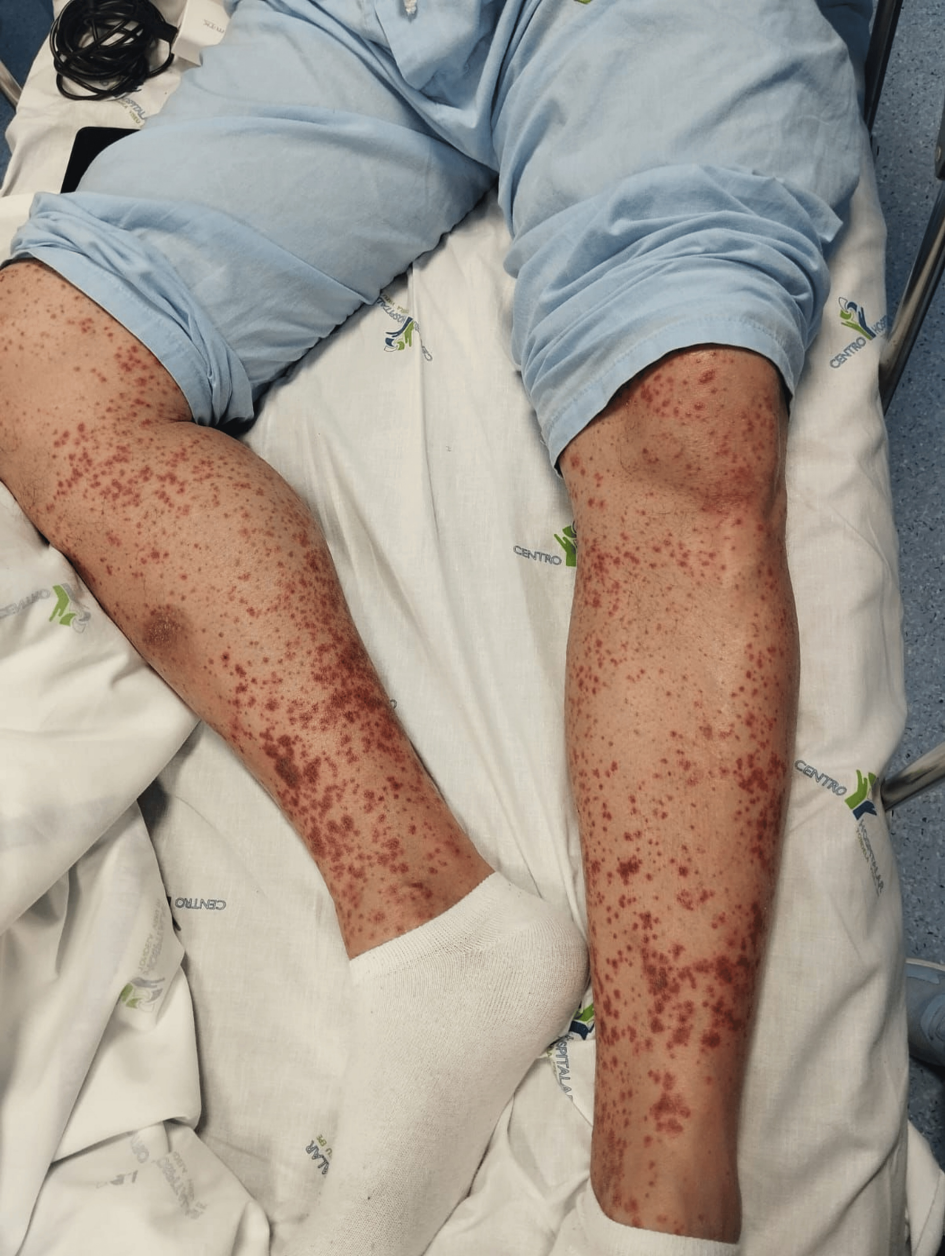
Palpable purpura on the lower limbs of the patient at admission

Our patient is a 41-year-old Caucasian man who is a non-smoker, independent in daily activities, with a personal history of obesity and a recent diagnosis of heart failure with reduced ejection fraction. The patient was medicated with pantoprazole 20 mg id, furosemide 40 mg id, lisinopril 5 mg id, bisoprolol 5 mg id, empagliflozin 10 mg id, spironolactone 50 mg id, and allopurinol 300 mg id about three days prior to the onset of lesions. He was admitted to the emergency department due to purpuric and pruritic lesions with preferential distribution on the lower limbs and subsequent cephalic progression with eight days of evolution (Figure 1) associated with constant epigastric pain without irradiation or aggravation/relief, factors lasting for six days. Anorexia, weight loss, syncope, genitourinary or osteoarticular changes, as well as symptoms suggestive of autoimmune pathology, were refuted.

**Figure 2:**
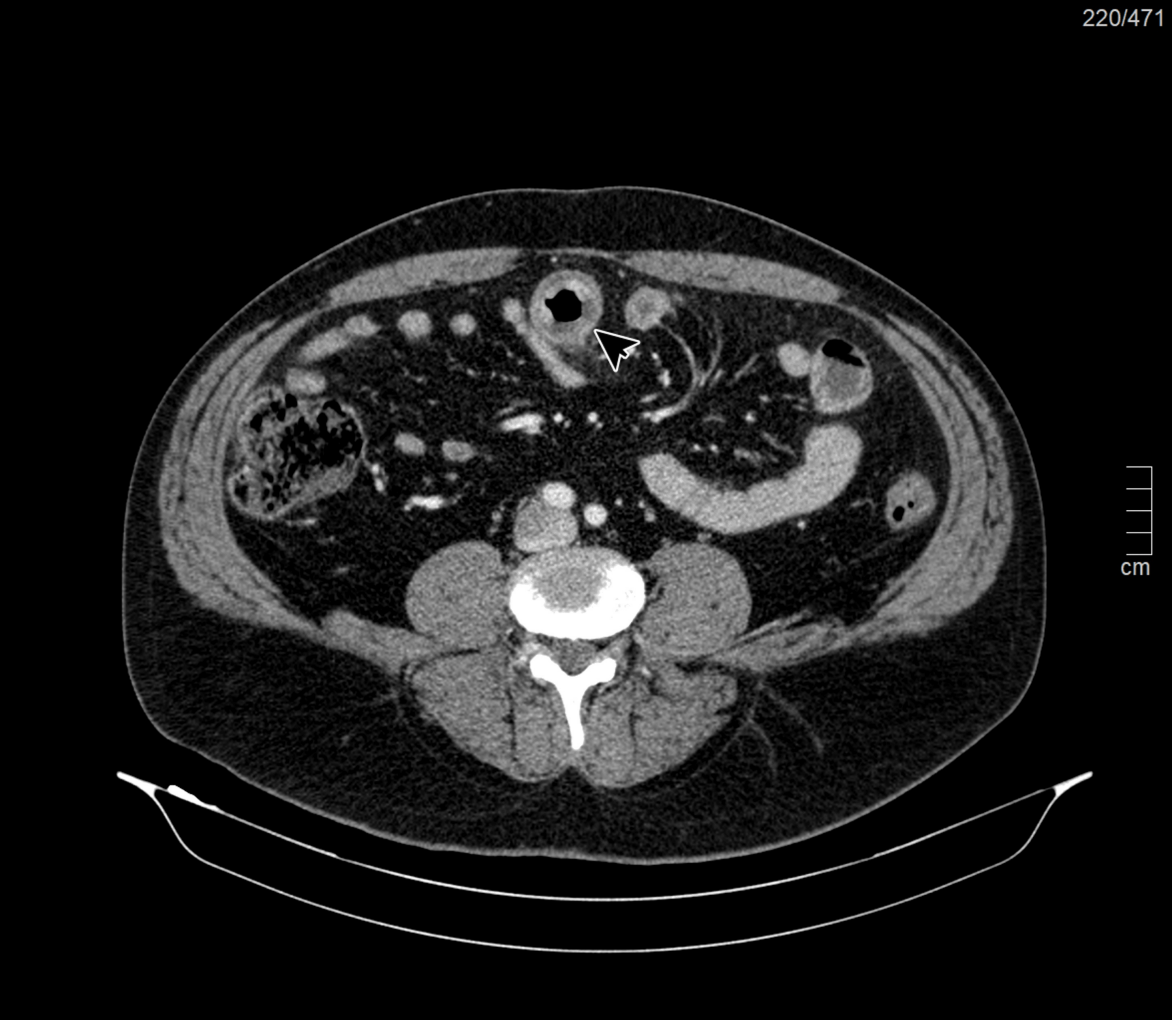
Axial plane of abdominal CT Marked circumferential thickening of the jejunal mucosa is noted with densification of the adjacent peritoneal fat and mesenteric vascular engorgement (arrowhead).

**Figure 3:**
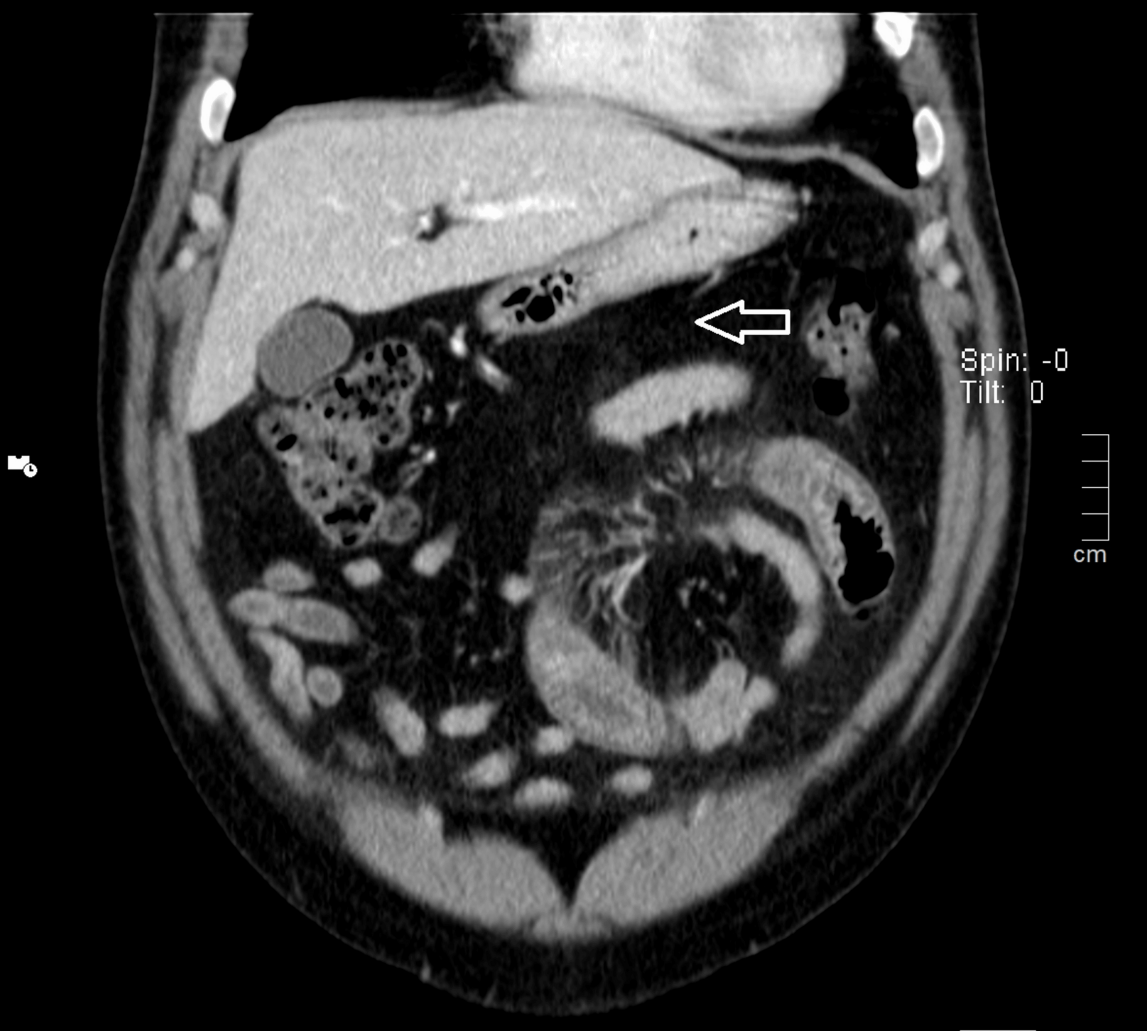
Coronal plane of abdominal CT Circumferential thickening of the jejunal mucosa measuring 5 inches to 9 inches in length is seen associated with densification of the adjacent tissues (arrow).

**Figure 4:**
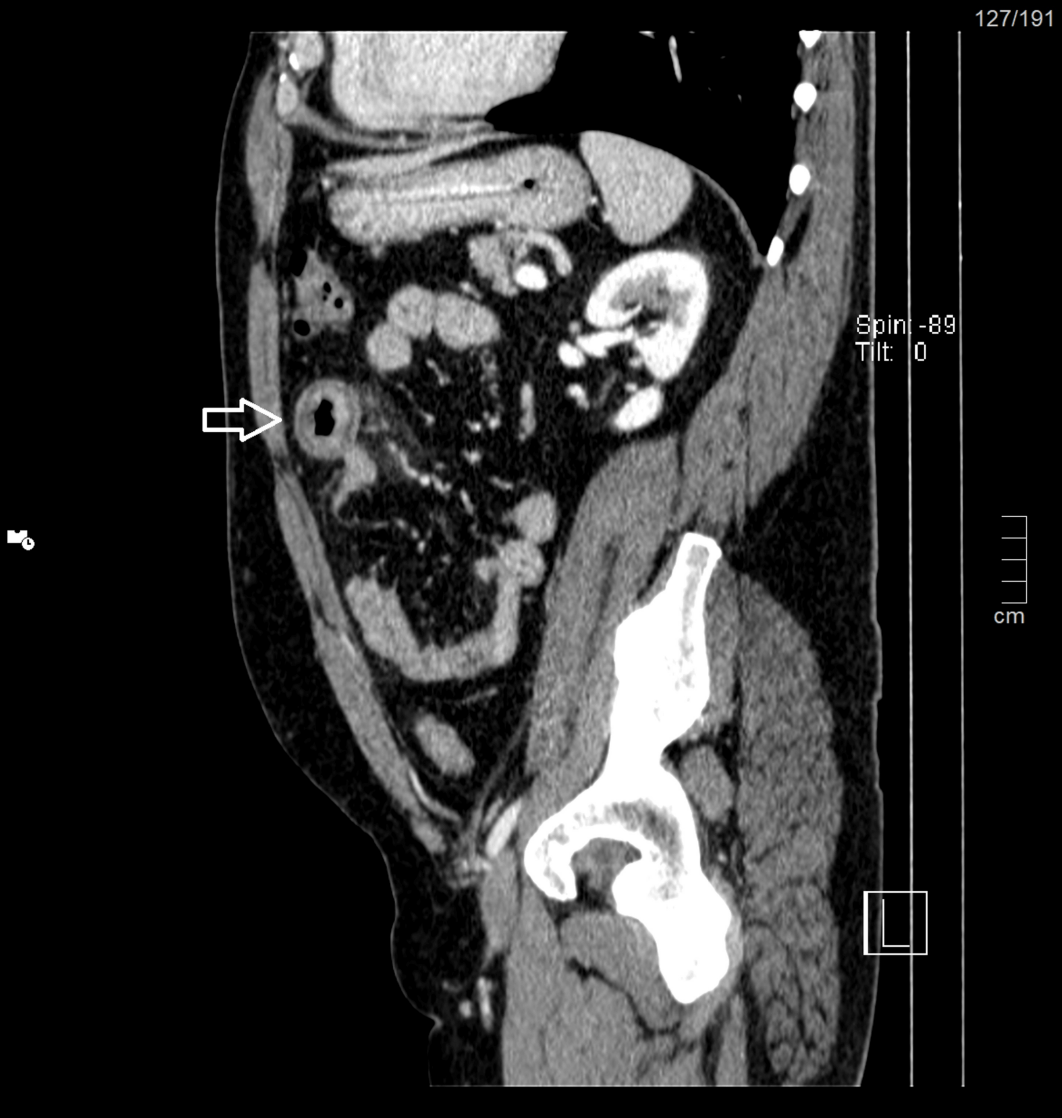
Sagittal plane of abdominal CT The inflammatory process of the jejunal mucosa with densification of the adjacent peritoneal fat and mesenteric vascular engorgement is observed (arrow).

**Figure 5:**
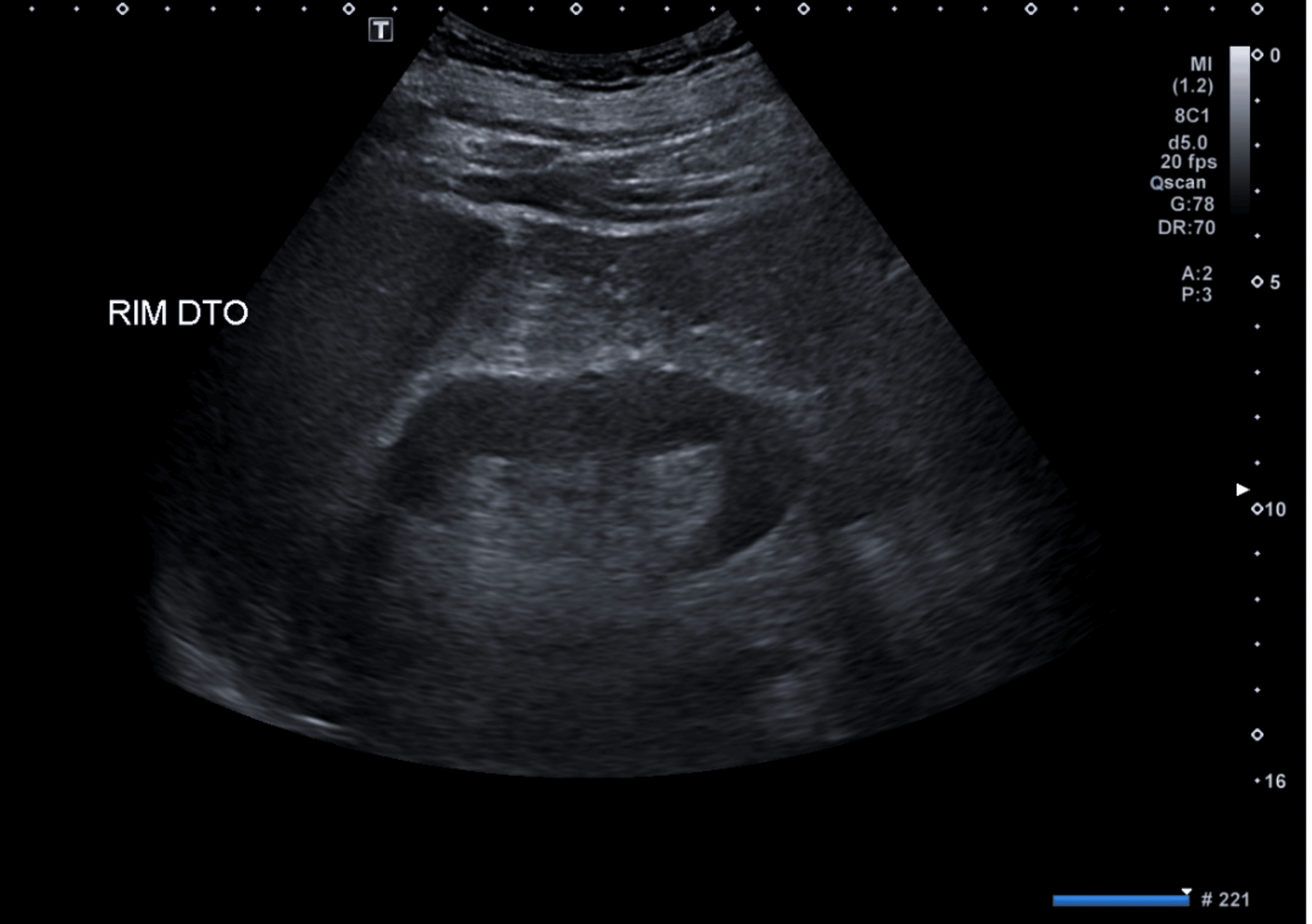
Right kidney ultrasound The kidneys were normal-sized and without morphological changes, showing normal parenchymal-sinus differentiation and no dilation of the renal excretory cavities on the right kidney.

**Figure 6:**
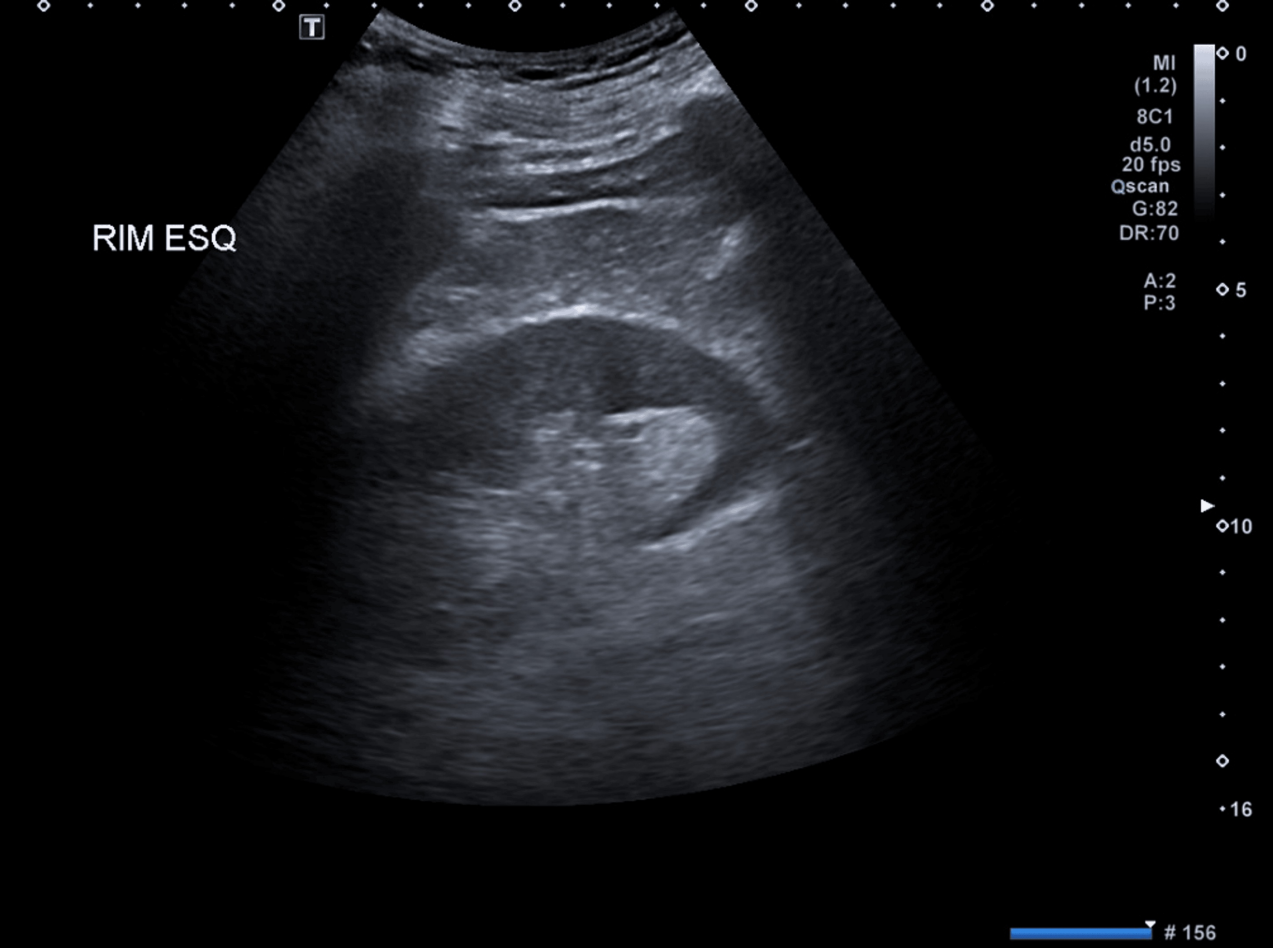
Left kidney ultrasound No changes were observed on this ultrasound.

The highlights of the complementary study of the patient carried out on admission (Table 1) are the absence of thrombocytopenia, no evidence of hemolysis or coagulopathy, no changes of renal function and ionogram, the absence of rhabdomyolysis, and the presence of microscopic hematuria in the summary urine study. Given the complaints of abdominal pain, an abdominal CT scan was performed with evidence of a marked circumferential thickening of the jejunal mucosa measuring 5 inches to 9 inches in length associated with densification of the adjacent peritoneal fat and mesenteric vascular engorgement inflammatory process translators (Figures 2-4). Renovesical ultrasound was performed, with no renal morphofunctional anomalies, i.e., normal-sized and well-differentiated kidneys with normal thickness of the renal parenchyma and without images translating an expansive lesion nor dilation of the excretory cavities (Figures 5-6).

**Table 1:** Results of complementary diagnostic exams performed SV: Sedimentation rate, LDH: Lactate dehydrogenase, uACR: Urine albumin-creatinine ratio

Analytical parameters	Results	References
Leukocytes	11.66 x 10^9/L	4.5-11.5 x 10^9/L
Hemoglobin	15.2 g/dl	14-18 g/dl
Platelets	216000 x 10^9/L	150-450 x 10^9/L
Prothrombin time	12.5 sec	11-14 sec
SV	1.0 mm	8-16 mm
Creatinine	0.9 mg/dl	0.5-1.2 mg/dl
LDH	216 UI/L	120-246 UI/L
uACR (urine sediment)	0.280	1.0-10.0
Erythrocyturia	31.7/uL	1.0-24.0/uL

During his stay in the emergency department, a dermatology consultation was required. Considering the given clinical findings, objective examination, and complementary tests, HSP was assumed as the most likely diagnostic hypothesis with an indication for hospitalization. An additional study was aimed at confirming clinical suspicion. 

**Figure 7:**
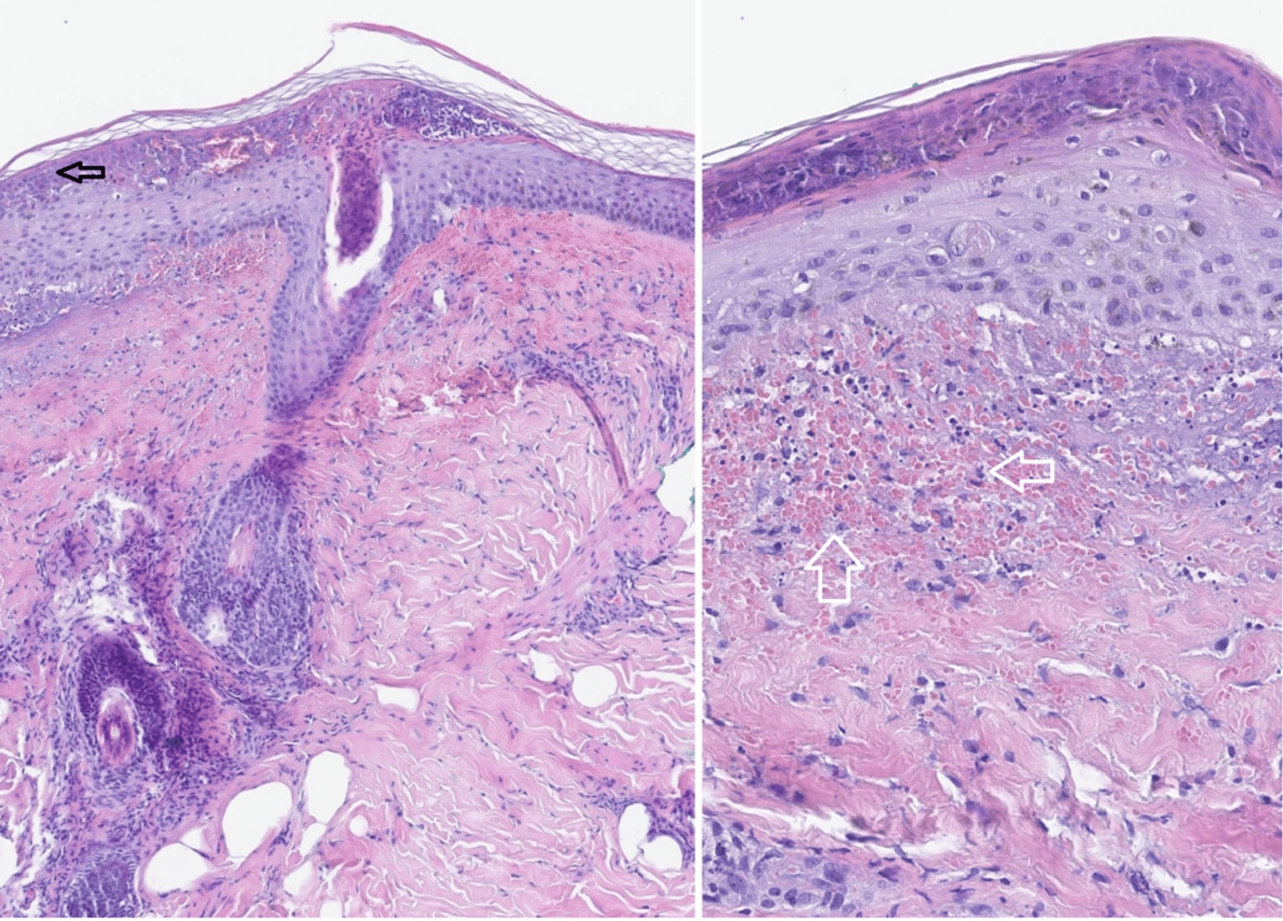
Skin biopsy of purpuric lesions Optical microscopy shows partial epidermal necrosis (black arrow on the left) and dermal invasion by red blood cells (white arrows on the right).

After four days of treatment with prednisolone (1 mg/kg per day), a regression of abdominal complaints and skin lesions on the trunk and upper limbs was verified with no abnormalities in urinary output or macroscopic characteristics of the urine. The patient was then evaluated by dermatology with a skin biopsy taken for optical microscopy and immunofluorescence studies. The anatomopathological result revealed a lesion evolving from leukocytoclastic vasculitis, with most of the findings in the immunofluorescence examination being nonspecific, although the discrete disposition of IgA in the superficial capillaries fit into the clinical hypothesis of HSP (Figure 7).

The autoimmune study was negative, including antinuclear antibodies, rheumatoid factor, extractable nuclear antigen (ENA), anti-*Saccharomyces cerevisiae* antibodies (ASCA), and anti-citrullinated protein antibodies, and immunoglobulin levels were also normal. The patient was immune to cytomegalovirus and Epstein Barr virus, with no reactivity in serological tests for toxoplasma, syphilis, HIV, and hepatitis B and C. Thyroid function and electrophoretic proteinogram were normal too.

Considering the normality of the complementary study carried out excluding immunological pathology, the possibility of an iatrogenic reaction to lisinopril introduced into the patient's therapeutic schedule three days before the onset of the clinical condition was assumed. On the sixth day of hospitalization, the patient was also evaluated by nephrology with the suggestion of the possibility of IgA nephropathy given the microscopic hematuria and A2 proteinuria with the suggestion of follow-up in a specialist consultation. 

The patient maintained apyrexia, hemodynamic, and clinical stability throughout his hospitalization. On the date of discharge, the patient was referred to an external internal medicine consultation (with a corticosteroid weaning regimen) and nephrology. Upon reassessment of the patient at the internal medicine consultation and while still in the weaning phase from corticosteroid therapy and the suspension of lisinopril, complete regression of the skin lesions was observed with no evidence of new clinical changes.

## Discussion

While a rare and challenging diagnosis, HSP may be suggested after applying the European Alliance of Associations for Rheumatology (EULAR) 2010 criteria. With a diagnostic sensitivity of 100% and specificity of 87%, the combination of purpuric lesions with at least one of the following four clinical criteria and histopathological findings (abdominal pain, polyarthralgia, renal involvement, and leukocytoclastic vasculitis/proliferative glomerulonephritis with perivascular IgA deposits) suggests this vasculitis as the most likely diagnostic hypothesis for our patient [3]. Although characteristic leukocytoclastic vasculitis does not represent a pathognomonic finding of HSP, it can be detected in hypersensitivity vasculitis secondary to B and C hepatitis infections and those associated with connective tissue diseases such as lupus [1].

In this presented case, the complementary study carried out in the hospital made it possible to exclude infectious and autoimmune etiologies in the genesis of the clinical changes presented, plus there were no recent immunizations nor signs and symptoms compatible with occult neoplastic disease. The knowledge of skin reactions as the main complication of ACE inhibitors as well as the reports of previous cases of HSP associated with these drugs used in hypertension and heart failure was decisive in the presumptive diagnosis of associated HSP to lisinopril, leading to its suspension on the fourth day of hospitalization [6-8]. The application of the Naranjo scale (probability scale of adverse drug reactions) to our clinical case classifies the diagnostic hypothesis as probable (7 points), i.e., the existence of a leukocyte transformation test in the presence of lisinopril or the recurrence of clinical manifestations when reintroduction of the drug would reinforce the degree of certainty of the clinical presumption [9-13].

Although the clinical manifestations are like those observed in pediatric age, in terms of prognosis the scenario is different, as only 20% of adults achieve complete remission after the initial diagnosis within a follow-up period of 15 years [3]. In around 20% of adults, recurrent systemic vasculitis occurs with a predominance of extra-renal clinical manifestations, with predictive factors being age over 30 years at the time of diagnosis, the presence of persistent skin lesions, abdominal pain, and/or hematuria. In adulthood, progression to chronic kidney disease is observed in 18% of patients and appears as the most feared complication. The existence of arterial hypertension, macroscopic hematuria, decreased glomerular filtration rate, and proteinuria greater than 1 g/24 h determine a worse prognosis [1,3]. Recent studies suggest measuring urinary IgA as the best predictor of renal prognosis in the first year after HSP diagnosis [5].

As observed in the diagnosis of vasculitis, there is also no consensual data regarding the therapeutic approach to HSP. In addition to discontinuation exposure to the trigger (a drug in our case) and supportive measures, the use of systemic corticosteroids, alone or in combination with rituximab or mycophenolate mofetil (promising results in recent studies), has an impact on controlling the progression of skin lesions, as well as the evolution of proteinuria and kidney disease [3,5]. In our clinical case, the extent and severity of the skin lesions associated with the presence of hematuria were decisive in the introduction of corticosteroid therapy into the patient's treatment regimen.

## Conclusions

Henoch-Schönlein purpura constitutes a challenging clinical entity both in terms of diagnostic approach and therapeutic intervention. Despite the low associated lethality and self-limited course of clinical manifestations in most patients, after the acute phase, where gastrointestinal complications represent the greatest threat, the long-term prognosis is determined by the extent of renal involvement, which is why monitoring patients in nephrology consultations seems essential to us. In this clinical case, in addition to the diagnostic process carried out, we intend to reinforce that cutaneous complications are one of the main adverse reactions in treatment with ACE inhibitors, which are drugs widely prescribed in our daily clinical activity.
